# Exploration, explanation and exploitation of hydroxyls in zeolites

**DOI:** 10.1093/nsr/nwac081

**Published:** 2022-04-29

**Authors:** Eddy Dib, Edwin B Clatworthy, Hugo Cruchade, Izabel C Medeiros-Costa, Nikolai Nesterenko, Jean-Pierre Gilson, Svetlana Mintova

**Affiliations:** Laboratory of Catalysis and Spectrochemistry (LCS), Normandy University, National Graduate School of Engineering of Caen (ENSICAEN), French National Center for Scientific Research (CNRS), France; Laboratory of Catalysis and Spectrochemistry (LCS), Normandy University, National Graduate School of Engineering of Caen (ENSICAEN), French National Center for Scientific Research (CNRS), France; Laboratory of Catalysis and Spectrochemistry (LCS), Normandy University, National Graduate School of Engineering of Caen (ENSICAEN), French National Center for Scientific Research (CNRS), France; Total Energies Research and Technology, Belgium; Total Energies Research and Technology, Belgium; Laboratory of Catalysis and Spectrochemistry (LCS), Normandy University, National Graduate School of Engineering of Caen (ENSICAEN), French National Center for Scientific Research (CNRS), France; Laboratory of Catalysis and Spectrochemistry (LCS), Normandy University, National Graduate School of Engineering of Caen (ENSICAEN), French National Center for Scientific Research (CNRS), France

## Abstract

The precise location and role of all types of hydroxyls in zeolites are still enigmatic, and their control permits tailoring of novel properties increasing the efficiency of catalysts and adsorbents in industrial processes for cleaner energy.

The ideal crystalline framework of zeolites is perturbed by hydroxyl groups. In pure silica zeolites the framework is neutral, however, the introduction of Al^3+^ into the Si^4+^ oxide framework creates negative charges, giving rise to Brønsted acid sites (BASs). Often referred to as bridged hydroxyls [Si–O(H)–Al], they are formed when a proton is bound to one of the oxygens adjacent to an aluminum. This hydroxyl group is at the heart of the remarkable properties of zeolites in acid-catalyzed reactions, as these sites and the reactants are confined in a well-defined molecule-sized environment. Other hydroxyl groups called silanols [Si–O(H)] are also present in the zeolite structure and their role is still not fully understood. Both the BAS and silanols are considered as defects of zeolite framework structures yet they hold the key to transforming inert perfect pure-silica frameworks such as quartz into chemically active materials.

BAS and silanols have been investigated for decades, during which time different characterization methods were employed in order to optimize the properties of zeolite-based catalysts, and will continue to intrigue researchers for years [1].

Various direct and post-synthesis approaches have been developed either to create or heal BAS or silanol groups in zeolites. Synthetic control of the distribution of Al in high-silica zeolites is critical due to the enormous number of Al arrangements and their effect on the catalytic performance of zeolites [2]. The distribution of both isolated and pairs of Al can be tuned by changing the polarization of the structure directing agent (SDA) or by varying the type of Al source. However, the combined use of inorganic and organic SDAs to achieve high-Al-containing zeolites creates a new challenge due to the apparent detrimental effect of other cations on the formation of Al pairs. Optimizing the combination of alkali metal cations and organic cationic SDAs for obtaining new zeolites will be crucial for controlling the Al site distribution and performance of new zeolite catalysts by controlling the bridged hydroxyls.

The main techniques used to characterize hydroxyls in zeolites are X-ray diffraction and infrared (IR) and nuclear magnetic resonance (NMR) spectroscopy [3]. Theoretical calculations have also provided a new tool for precise assignments [4]. Their combination provides a complete vision of the distribution of these sites, as each technique highlights a specific feature. However, each technique has the intrinsic weaknesses of low resolution and sensitivity. Various classifications of the hydrogen bonds have been suggested based on their strength, assessed in terms of binding energy, hydrogen bonding distance, elongation of the bond, etc. Emerging and powerful new electron diffraction methodologies are now available to locate hydrogen atoms, and their development will likely bring rich structural information on hydroxyl groups [5].

Silanols present a different but equally important type of hydroxyl group because they can act as sites for further functionalization, i.e. introducing metals into the zeolite framework [6]. Post-synthetic strategies typically involve the initial formation of silanol groups by acidic dealumination treatments followed by metal incorporation, however, this can lead to incomplete removal of isolated silanols. Recent efforts have shown that metals can be incorporated into zeolite frameworks using direct synthesis strategies resulting in the complete healing of the hydroxyl sites. Different factors, including the presence of alkali metal cations, and the type of silanols, are key in determining whether metals are incorporated into the framework [7]. The great challenge is the incorporation of a higher number of metals, understanding the mechanism of catalyst deactivation, and catalyst stabilization. Single-metal-site catalysts with high stability, accessibility and controlled distribution of hydroxyls (BAS and silanols) are of critical importance.

The properties of BAS are one of the major concerns when it comes to designing zeolitic catalysts [7]. Regarding the local environment, the interaction of an acidic proton and an extra-framework aluminum may enhance the alkane cracking activity. Furthermore, the zeolite local environment plays a crucial role in activity and selectivity by stabilizing

 

transitional states of reactive intermediates in the confined void; this is described as the solvation effect [8]. At relatively longer range the aluminum distribution and the size of the crystal affect the acidity of the zeolite. Hydrogen mobility over the zeolitic crystal or neighboring active sites is another important consideration for reactions such as hydrogenation or for other applications including gas storage, purification and separation. Thus, the BAS activity must be considered a combination of interactions established at different length scales; deciphering the relationship between BAS properties and the catalytic behavior of zeolites is an ongoing challenge [9].

Besides acidic activity, hydroxyl groups play a key role in bifunctional catalysts by providing anchor points to disperse metals [1].

The understanding of hydroxyls in acidic catalysts is continuously progressing; the constant evolution of synthesis and characterization methods will undoubtedly reveal more about the role of silanols in catalysis and allow tuning of new active sites. For example, Lewis Acidic sites possessing hydroxyl groups may arise from the formation of extra-framework Al species that can

exist in different oxide and hydroxide forms [10]. Still, the challenging questions are how to control hydroxyls in zeolites using different synthesis methodologies and how to identify them and relate them to the reactivity of zeolites. A specific challenge is the characterization of hydroxyls in zeolites with atomic resolutions via *in situ* and operando techniques. Considering the above statements, several additional questions need to be answered: how do silanols impact the local and extended environments of BAS? Is there a synergetic effect between silanols and neighboring BAS upon activity or hydrogen mobility?

From this, we can clearly see that hydroxyls in zeolites demand further understanding. They belong to the ‘Zeolite Crystal Engineering’ toolbox for designing new materials for catalysis and adsorbents. Advances in the understanding of hydroxyls in zeolites will provide endless opportunities for discovering zeolites with original properties not previously known (Fig. 1). In the long term, zeolites with controlled hydroxyls (BAS and silanols) will contribute to society by addressing the challenges of energy transition and climate change, and provide solutions to reduce CO_2_ emissions.

**Figure 1.**
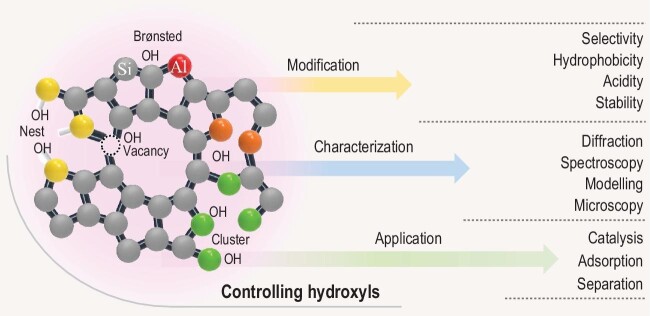
Exploration, explanation and exploitation of hydroxyls with the aim of creating zeolites with novel properties.
